# CD30 expression in diffuse large B-cell lymphoma and primary mediastinal lymphoma: frequency, association with prognostic factors, and impact on survival

**DOI:** 10.1016/j.htct.2026.106493

**Published:** 2026-08-05

**Authors:** Marcelo Bellesso, Ibere Cauduro Soares, Frederico Rafael Moreira, Juliana Pereira, Vanderson Rocha

**Affiliations:** Universidade de Sao Paulo Instituto do Cancer do Estado de Sao Paulo, São Paulo, SP, Brazil

**Keywords:** Diffuse large B cell lymphoma, Primary mediastinal B-cell lymphoma, CD30 expression

## Abstract

**Introduction:**

CD30 expression has been studied as a prognostic and therapeutic biomarker in diffuse large B-cell lymphoma not otherwise specified (DLBCL-NOS) and primary mediastinal B-cell lymphoma (PMBCL). This study evaluated the frequency of CD30 expression, its association with National Comprehensive Cancer Network International Prognostic Index and cell of origin and impact prognosis.

**Methods:**

This retrospective cohort study included patients diagnosed with DLBCL or PMBCL between 2009 and 2016 at the Instituto do Câncer do Estado de São Paulo. Adult patients who received curative-intent treatment and had available histopathological material were included. CD30-positive cases were tested for Epstein-Barr virus by in situ hybridization: positive cases were excluded.

**Results:**

A total of 301 patients were analyzed (279 DLBCL and 22 PMBCL). CD30 positivity was found in 19.6% of cases (19% in DLBCL and 27.3% in PMBCL). Among the CD30-positive cases, nodal biopsies were more frequent (62.7%) than extranodal (37.3%) (p = 0.03). No significant association was found between CD30 expression and the National Comprehensive Cancer Network International Prognostic Index or cell of origin (germinal center B-cell-like vs. non-germinal center B-cell-like). CD30 expression did not impact overall survival (p = 0.22) or progression-free survival (p = 0.42). In the DLBCL subgroup, CD30 expression also showed no significant effect on overall survival (p = 0.35) or progression-free survival (p = 0.70).

**Conclusion:**

This is the first Brazilian and Latin American study to assess CD30 expression in DLBCL and PMBCL. CD30 expression was observed in 19.6% of cases, more commonly in nodal biopsies, but showed no prognostic significance.

## Introduction

Diffuse large B-cell lymphoma, Not Otherwise Specified (DLBCL-NOS) is the most common subtype of mature B-cell lymphoma, accounting for approximately 35% of cases in Western countries [1]. In Brazil, its incidence is even higher, representing nearly 50% of all non-Hodgkin lymphoma (NHL) cases [2]. Over the past two decades, therapeutic advances for DLBCL-NOS have been modest. The addition of rituximab to the standard CHOP regimen (cyclophosphamide, doxorubicin, vincristine, and prednisone) has become the least toxic and most effective first-line option [3, 4, 5].

Although rituximab-based therapy has improved survival outcomes, approximately 40% of patients still require salvage therapy [6,7]. More recently, the addition of polatuzumab vedotin — an antibody-drug conjugate targeting CD79b — plus R-CHP (rituximab, cyclophosphamide, doxorubicin, prednisone) demonstrated improved progression-free survival (PFS) compared to R-CHOP (rituximab plus CHOP), with a 27% reduction in the risk of progression or death (Hazard ratio [HR] = 0.73; 95% Confidence interval [95% CI]: 0.57–0.95; p = 0.020) [8].

The biological heterogeneity of DLBCL-NOS, which encompasses various lymphoproliferative disorders under the same diagnostic category, continues to drive efforts to refine its clinical, immunophenotypic, and molecular classification. These efforts aim to better understand disease pathophysiology and identify actionable therapeutic targets to develop more effective and less toxic treatments.

In 2008, primary mediastinal B-cell lymphoma (PMBCL) was recognized as a distinct clinicopathological entity, due to its unique thymic origin and clinical presentation [9]. This led to dedicated clinical trials, including the dose-adjusted R-EPOCH regimen (rituximab, etoposide, prednisone, vincristine, cyclophosphamide, and doxorubicin), which demonstrated favorable response rates and survival outcomes without requiring radiotherapy [10]. However, a recent retrospective study from Brazil found that survival outcomes for patients treated with R-CHOP (with or without etoposide) were comparable to those achieved with DA-R-EPOCH (Dose-Adjusted Rituximab, Etoposide, Prednisone, Oncovin, Cyclophosphamide, Hydroxydaunorubicin) [11].

CD30 plays a role in the generation and maintenance of memory B and T cells; furthermore, it is expressed in Epstein-Barr virus (EBV)-infected B cells and classic Hodgkin lymphoma [12,13], cutaneous T-cell lymphoma and anaplastic large cell lymphoma [14,15]. Its restricted expression profile has made it a promising therapeutic target. The anti-CD30 antibody-drug conjugate brentuximab vedotin, which delivers monomethyl auristatin E, has demonstrated efficacy in classic Hodgkin lymphoma [16, 17, 18, 19, 20, 21], anaplastic large cell lymphoma [22,23], and CD30-positive cutaneous T-cell lymphomas [24, 25, 26].

Studies assessing CD30 expression remain scarce in DLBCL-NOS, with reported expression rates ranging from 8%−41%. No consensus has been established regarding the optimal cutoff to define CD30 positivity, with thresholds varying between ≥1% and >20% [27, 28, 29, 30, 31, 32, 33, 34]. Furthermore, the prognostic significance of CD30 expression remains uncertain, as most studies have not evaluated correlations with established prognostic markers such as cell of origin or international prognostic indices [27,29, 30, 31, 32]. Furthermore, there is no published data on the Brazilian and Latin American population regarding CD30 expression.

Accordingly, this study aimed to determine the frequency of CD30 expression in DLBCL-NOS and PMBCL and to explore its prognostic impact. In addition, the associations between CD30 expression, the National Comprehensive Cancer Network International Prognostic Index (NCCN-IPI), and the cell of origin were investigated.

## Methods

This retrospective observational cohort study was approved by the Research Ethics Committee of the Cancer Institute of the State of São Paulo (ICESP) in May 2017. Patients, identified from the ICESP non-Hodgkin lymphoma outpatient clinic database, were included if they were diagnosed between January 1, 2009 and December 31, 2016. Eligible individuals were aged ≥18 years, had a confirmed diagnosis of DLBCL-NOS or PMBCL, received curative-intent treatment with R-CHOP, R-CHOEP, or DA-R-EPOCH, and had available formalin-fixed, paraffin-embedded tissue blocks suitable for histopathological analysis. Exclusion criteria comprised AIDS-related lymphomas, or DLBCL with primary central nervous system (CNS) involvement, or CD30 expression >0% in association with EBV positivity as determined by in situ hybridization. EBV positivity was defined as the presence of one or more tumor cells showing positive staining by in situ hybridization.

**Figure 1:**
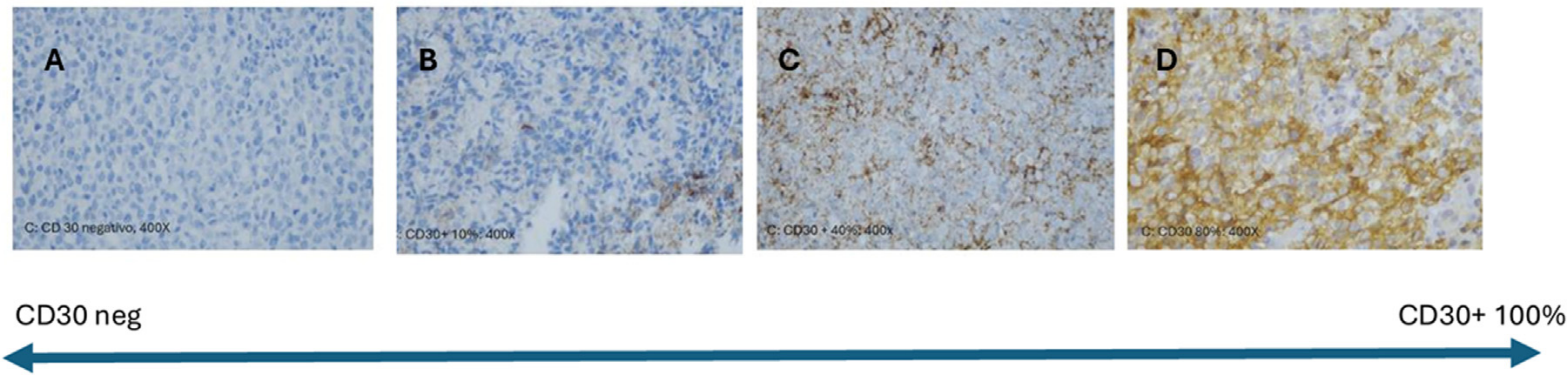
Tumor tissue sections from four distinct cases at 400× magnification, representing the spectrum of CD30 expression using the CD30 (Ber-H2) reagent. A: absence of CD30 expression; B: 10% CD30 expression; C: 40% CD30 expression; D: 80% CD30 expression.

All cases were reviewed by a hematopathologist. The same immunohistochemical reagents and primary antibodies (clones, Ventana) were used throughout the entire analysis period: CD20 (L26), CD10 (SP67), BCL6 (GL19E/A8), MUM1 (MRQ-43), MYC (Y69), BCL2 (124), and CD30 (Ber-H2). CD30 expression was assessed in CD20-positive neoplastic cells and categorized as negative (0%) or positive (0–20%, 20–40%, >40%) (Figure 1). EBV status was assessed for CD30-positive cases. Diffuse large B-cell lymphomas were subclassified as germinal center B-cell-like (GCB) or non-GCB using the Hans algorithm, utilizing a 30% cut-off for CD10, BCL6, and MUM1 expression [35].

Clinical, epidemiological, and laboratory data were retrieved from electronic medical records and the HCMED platform. Disease staging followed the Lugano 2014 Positron Emission Tomography–Computed Tomography criteria [36]. Prognostic stratification was based on the Revised International Prognostic Index (R-IPI) [7] and the NCCN-IPI [37].

Response was assessed using Lugano 2014 criteria [36]. PFS was defined as the time from diagnosis to relapse, progression, or death. Overall survival (OS) was defined as the time from diagnosis to death from any cause. Patients without events were censored at the last follow-up.

### Statistical analysis

Survival was analyzed using the Kaplan–Meier method, with differences assessed by the log-rank test [38]. Associations between covariates and time-to-event outcomes were evaluated using univariate Cox regression; variables with p-value <0.15 were included in a multivariable Cox model and selected via backward elimination (α = 0.05) to obtain independent predictors [39,40]. Multicollinearity was assessed using variance inflation factors (VIFs). To mitigate collinearity, any covariate exhibiting a VIF ≥2.5 that held lesser clinical relevance was excluded from the backward selection process [41]. Continuous covariates were dichotomized using either clinically meaningful cut-off values or optimal cut-off points determined by the Contal and O’Quigley method [42,43]. The proportional hazards assumption was checked using a Kolmogorov-type supremum test and weighted Schoenfeld residuals [44,45]. Categorical variables were compared using chi-square or Fisher’s exact tests [46], and continuous variables were summarized as medians and interquartile ranges (IQR) if not normally distributed [46]. Normality was assessed by visual inspection of histograms and the Shapiro–Wilk test [47,48]. Median follow-up was calculated using the reverse Kaplan–Meier method. Analyses were performed using SAS 9.4 (SAS Institute Inc., Cary, NC), and two-sided p-values <0.05 were considered statistically significant.

## Results

**Figure 2:**
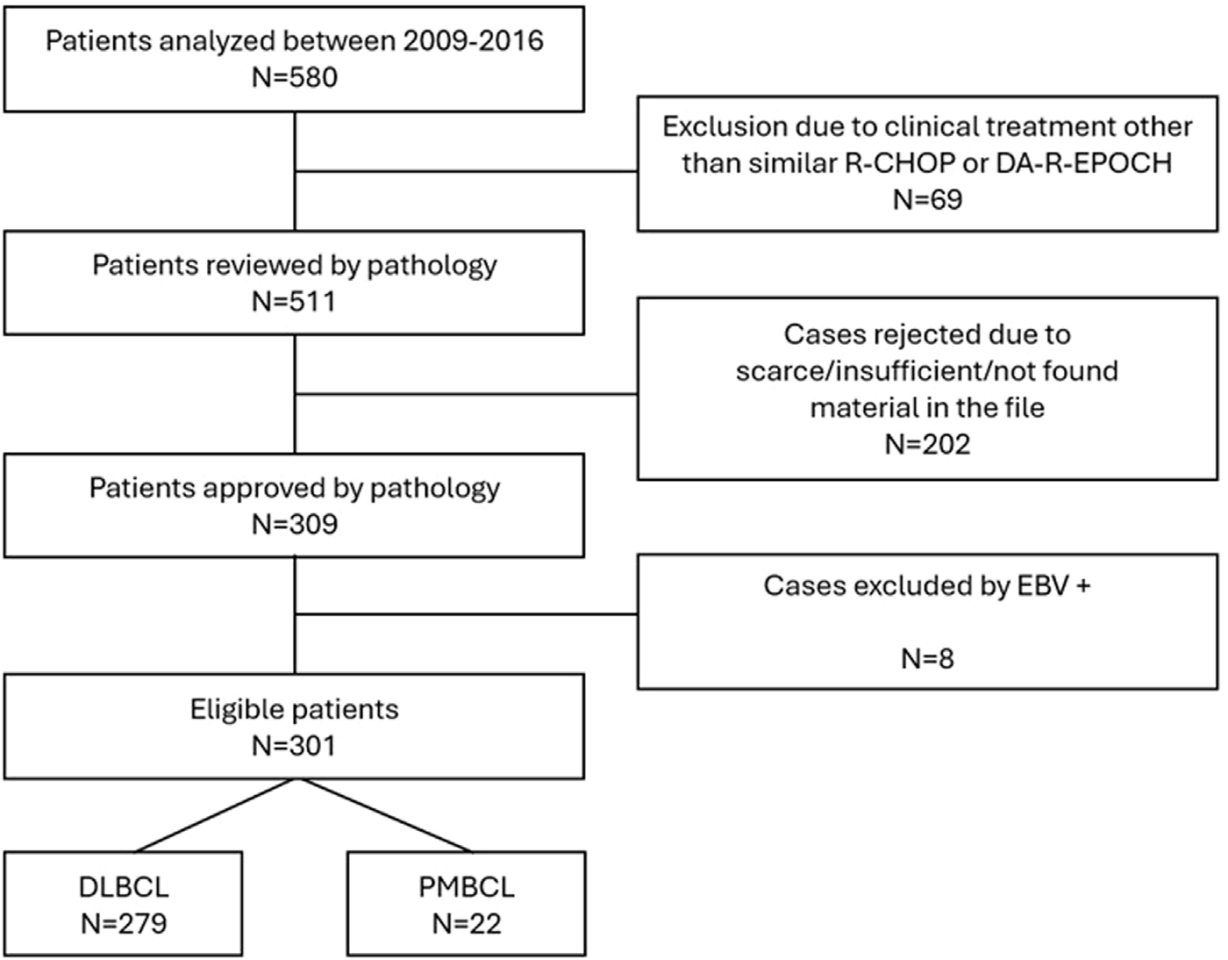
Eligibility criteria and case screening process.

A total of 580 patients, diagnosed with DLBCL-NOS and PMBCL between January 2009 and December 2016, was evaluated. Of these, 301 cases were eligible: 279 (92.7%) were diagnosed with DLBCL and 22 (7.3%) with PMBCL (Figure 2). The clinical parameters, risk factors, and treatment strategies are summarized in Table 1.

**Table 1:** Distribution of clinical and diagnostic parameters, risk factors, and treatment.

	Total n = 301	DLBCL-NOS n = 279	PMBCL n = 22
Female - n (%)	153 (50.8)	143 (51.3)	10 (45.5)
Median age – median (IQR)	58.4 (42.6–66.7)	59.3 (45.2–68.2)	32.4 (27- 48.5)
Subtype - n (%)			
DLBCL-NOS	279 (92.7)	279 (100)	-
PMBCL	22 (7.3)	-	22(100)
B symptoms - n (%)	208 (69.1)	189 (68.5)	19 (86.4)
Staging - n (%)			
I	20 (6.6)	15 (5.4)	5 (22.7)
II	69 (22.9)	61 (21.9)	8 (36.3)
III	40 (13.3)	40 (14)	-
IV Missing data	170 (56.4) 2	162 (58.6) 1	8 (36.3) 1
ECOG - n (%)			
0	120 (39.8)	111 (39.7)	9 (40.9)
1	103 (34.2)	93 (33.5)	10 (45.5)
2	36 (12.8)	35 (12.6)	1 (4.5)
≥ 3	40 (13.2)	38 (13.7)	2 (9.1)
Missing data	2	2	-
Extranodal involvement - n (%)	250 (83)	238 (85.3)	12 (54.5)
Elevated LDH - n (%)	179 (59.4)	162 (58)	17 (77.2)
NCCN-IPI score - n (%)			
0–1	39 (12.9)	32 (11.5)	7 (31.8)
2–3	105 (34.9)	97 (34.7)	8 (36.4)
4–5	135 (44.9)	128 (45.8)	7 (31.8)
≥ 6	22 (7.3)	22 (7.9)	-
R-IPI score - n (%)			
0	30 (9.9)	27 (9.7)	3 (13.6)
1–2	116 (38.4)	102 (36.6)	14 (63.7)
≥ 3	155 (51.8)	150 (53.7)	5 (22.7)
Treatment - n (%)			
R-CHOP/ RCHOEP	272 (90.5)	259 (92.8)	13 (59)
DA-R-EPOCH	6 (2)	1 (0.4)	5 (22.7)
R-CHOMP	21 (6.9)	17 (6.1)	4 (18.3)
Others*	2 (0.6)	2 (0.7)	-

⁎ Cytoreductive treatment with COP regimen with therapeutic intent, followed by disease progression and death. DLBCL-NOS: Diffuse large B-cell lymphoma not otherwise specified; PMBCL: primary mediastinal B-cell lymphoma; IQR: Interquartile range; ECOG: Eastern Cooperative Oncology Group; LDH: lactate dehydrogenase; NCCN-IPI: National Comprehensive Cancer Network International Prognostic Index; R-IPI: Revised International Prognostic Index.

Among the 301 cases, 59 (19.6%) exhibited CD30 expression; of these, 50.8% (n = 30) demonstrated expression in >40% of the evaluated tumor tissue. The median CD30 expression in neoplastic tissue was 50%, ranging from 5%−100%. The distribution according to CD30 expression strata was as follows: ≤20% expression in 23 cases (7.6%), 21%−40% expression in six cases (2.0%), and >40% expression in 30 cases (10.0%) (Table 2). Due to the small number of CD30-positive cases, establishing an optimal cutoff value was not feasible. Therefore, any CD30 expression (>0%) was utilized as the threshold for prognostic evaluation. An assessment of clinical associations and prognostic factors within the PMBCL subgroup (n = 22) was not feasible due to the limited sample size. Regarding cell-of-origin classification, 141 cases (46.8%) were categorized as the GCB phenotype according to the Hans algorithm (Table 3). Only four cases exhibited concurrent expression of BCL2 and MYC; consequently, due to this limited sample size, statistical analysis of this subgroup was not feasible.

**Table 2:** Distribution of CD30 expression.

	Total (n = 301) n (%)	DLBCL-NOS (n = 279) n (%)	PMBCL (n = 22) n (%)
CD30-negative	242 (80.4)	226 (81)	16 (72.7)

CD30-positive	59 (19.6)	53 (19)	6 (27.3)
≤20%	23 (7.6)	22 (7.9)	1 (4.5)
20%−40%	6 (2)	6 (2.2)	-
>40%	30 (10)	25 (9)	5 (22.7)

DLBCL-NOS: Diffuse large B-cell lymphoma not otherwise specified; PMBCL: primary mediastinal B-cell lymphoma.

**Table 3:** Distribution of NCCN-IPI risk factor analysis, cell-of-origin and CD30 expression.

	CD30 expression		p-value
	0	>0%	
**DLBCL-NOS and PMBCL (n=301)**			
**NCCN-IPI - n (%)** Low risk (0–1) Low- Intermediate risk (2–3) High-Intermediate risk (4–5) High risk (≥ 6)	32 (82.1) 79 (75.2) 113 (83.7) 18 (81.8)	7 (17.9) 26 (24.8) 22 (16.3) 4 (18.2)	0.423^1^
**Cell-of-origin classification - n (%)**			0.634^1^
GC	115 (81.5)	26 (18.5)	
Non-GC	127 (75)	33 (25)	
**DLCBL -NOS (n=279)**			
**NCCN-IPI - n (%)** Low risk (0–1) Low- Intermediate risk (2–3) High-Intermediate risk (4–5) High risk (≥ 6)	26 (81.3) 74 (76.3) 108 (84.4) 18 (81.8)	6 (18.8) 23 (23.7) 20 (15.6) 4 (18.2)	0.495^2^
**Cell-of-origin classification - n (%)**			0.699^1^
GC	109 (81.9)	24 (18.1)	
Non-GC	117 (80.1)	29 (19.9)	

1 Chi-square test. ^2^ Fisher's exact test. DLBCL-NOS: Diffuse large B-cell lymphoma not otherwise specified; PMBCL: primary mediastinal B-cell lymphoma; NCCN-IPI: National Comprehensive Cancer Network International Prognostic Index; GC: Germinal center B-cell-like.

Regarding biopsy type, 134 samples (44.6%) were incisional, 79 (26.2%) were excisional, and 88 (29.2%) were core needle biopsies. No significant association was observed between CD30 expression and biopsy type (p = 0.69). However, CD30 expression differed significantly by anatomic site (p = 0.036). CD30-positive cases were more frequently observed in nodal biopsies (n = 37; 62.7%) than in extranodal biopsies (n = 22; 37.3%). Conversely, among CD30-negative cases, 115 (47.5%) were nodal and 127 (52.5%) were extranodal.

In both the overall cohort (n = 301) and the DLBCL-NOS subgroup (n = 279), CD30 expression was not significantly associated with cell-of-origin classification (GCB vs. non-GCB) or NCCN-IPI risk categories (Table 3).

**Figure 3:**
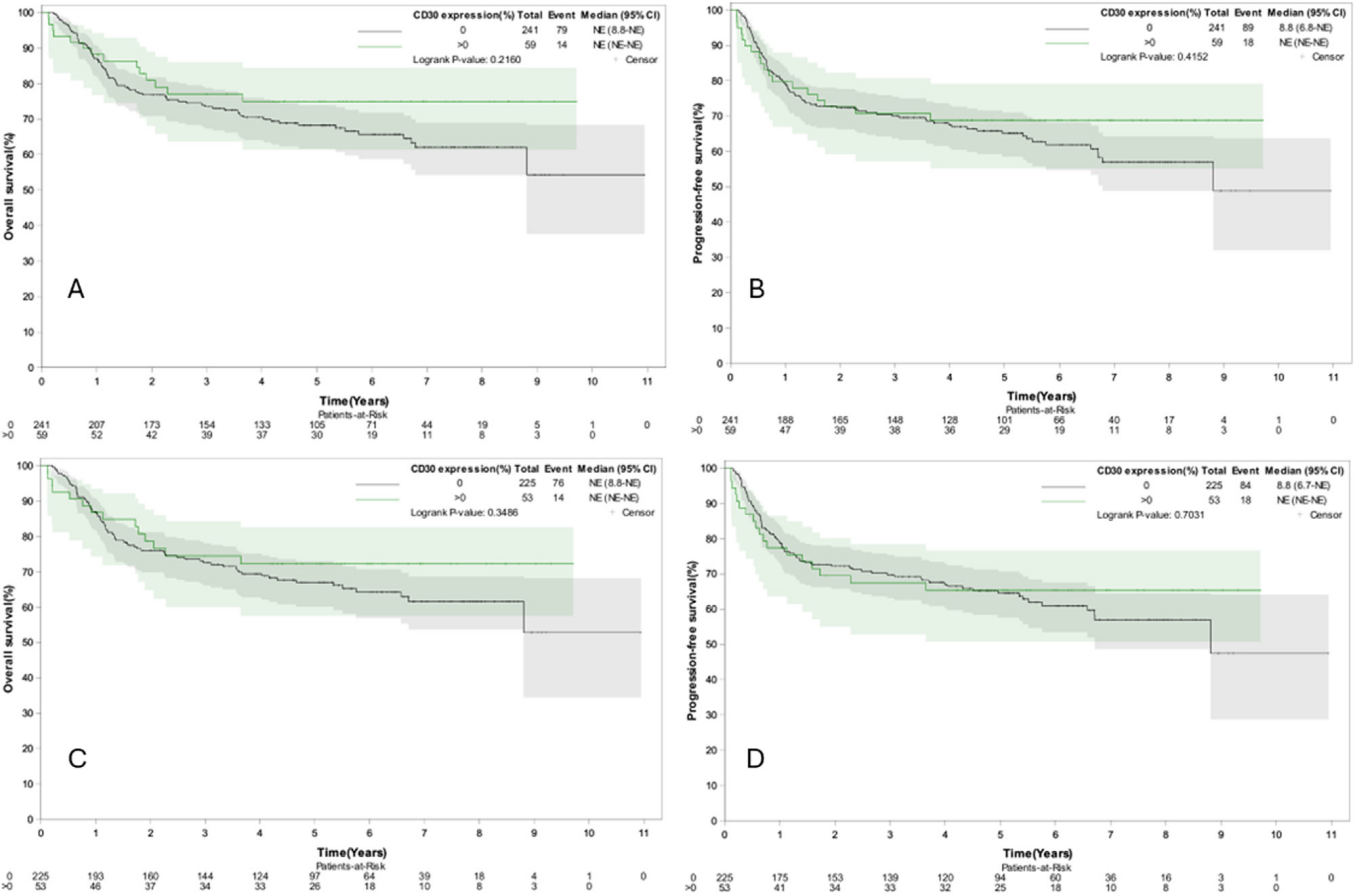
Kaplan-Meier survival curves for the study population. Panels A and C represent overall survival (OS), while panels B and D show progression-free survival (PFS). Panels A and B correspond to the overall cohort (n = 301), and panels C and D correspond to the DLBCL-NOS subgroup (n = 279).

The median OS was not reached at a median follow-up of 5.5 years (95% CI: 5.3–6.0). The 5-year OS rate was 69.6% (95% CI: 63.8–74.6%). The median PFS was also not reached, with a 5-year PFS rate of 65.9% (95% CI: 60.0–71.1%). Regarding CD30 expression, in the total cohort (n = 301), no differences were observed in OS or PFS between CD30-positive (>0%) and CD30-negative cases. For OS, the hazard ratio (HR) was 0.70 (95% CI: 0.40–1.24; p = 0.219), and for PFS, the HR was 0.81 (95% CI: 0.49–1.35; p = 0.417) (Figure 3). Due to the higher frequency of CD30 expression in nodal biopsies, an analysis was performed focusing only on the subgroup of cases with samples derived from nodal biopsies (n = 152). No differences were observed in OS or PFS in relation to CD30 expression. The HR for OS was 0.66 (95% CI: 0.32–1.36; p = 0.264), and for PFS, it was 0.62 (95% CI: 0.31–1.23; p = 0.17).

In DLBCL-NOS cases, no significant differences in OS or PFS were found according to CD30 expression status. The HR for OS was 0.76 (95% CI: 0.43–1.35; p = 0.35), and for PFS, the HR was 0.91 (95% CI: 0.54–1.51; p = 0.703) (Figure 3).

Significant differences in both OS and PFS were identified across the NCCN-IPI risk categories within the DLBCL-NOS cohort (p < 0.0001). The estimated 5-year OS rates were: low risk, 100.0%; low-intermediate risk, 79.0% (95% CI: 68.0–85.0%); high-intermediate risk, 56.0% (95% CI: 46.0–64.0%); and high risk, 45.0% (95% CI: 24.0–64.0%). Correspondingly, the estimated 5-year PFS rates were: low risk, 91% (95% CI: 78–99%); low-intermediate, 77% (95% CI: 67–84%); high-intermediate, 52% (95% CI: 43–61%); and high risk, 41% (95% CI: 21–60%; p < 0.0001).

In the univariable Cox regression analyses for the overall cohort, covariates with a p-value <0.15 were selected for multivariable modeling. For PFS, these candidates included age, B symptoms, Lugano stage, Eastern Cooperative Oncology Group performance status (ECOG-PS), lactate dehydrogenase (LDH) levels, NCCN-IPI, R-IPI, and treatment regimen (Supplementary Table 1). For OS, the candidate variables were lymphoma subtype, age, B symptoms, Lugano stage, ECOG-PS, LDH levels, NCCN-IPI, and R-IPI (Supplementary Table 3).

Within the DLBCL-NOS subgroup, several covariates met the univariable threshold for inclusion in the multivariable models. For PFS, these variables included age, B symptoms, Lugano stage, ECOG-PS, LDH levels, NCCN-IPI, and R-IPI (Supplementary Table 5). For OS, the candidate predictors were cell-of-origin classification, age, B symptoms, Lugano stage, ECOG-PS, LDH levels, NCCN-IPI, and R-IPI (Supplementary Table 7).

In the multivariable Cox regression analyses using backward selection, age >60 years, the presence of B symptoms, and advanced Lugano stage (III–IV) remained independently associated with a higher risk of both progression and death. For PFS, independent predictors included age (adjusted HR [aHR]: 1.70, 95% CI: 1.15–2.50; p = 0.007), B symptoms (aHR: 1.82, 95% CI: 1.12–2.97; p = 0.016), and Lugano stage (aHR: 2.04, 95% CI: 1.24–3.38; p = 0.005; Supplementary Table 2). For OS, independent predictors were age (aHR: 1.89, 95% CI: 1.24–2.87; p = 0.003), B symptoms (aHR: 1.75, 95% CI: 1.03–2.95; p = 0.037), and Lugano stage (aHR: 2.45, 95% CI: 1.38–4.36; p = 0.002; Supplementary Table 4)

Similarly, within the DLBCL-NOS subgroup, these same three covariates remained independent predictors of inferior outcomes. For PFS, independent risk factors included age (aHR: 1.57, 95% CI: 1.05–2.34; p = 0.026), B symptoms (aHR: 1.87, 95% CI: 1.14–3.08; p = 0.014), and advanced Lugano stage (aHR: 2.53, 95% CI: 1.43–4.47; p = 0.001; Supplementary Table 6). For OS, independent predictors were age (aHR: 1.74, 95% CI: 1.14–2.68; p = 0.011), B symptoms (aHR: 1.86, 95% CI: 1.08–3.18; p = 0.025), and advanced Lugano stage (aHR: 2.81, 95% CI: 1.48–5.33; p = 0.002; Supplementary Table 8). Notably, the NCCN-IPI was excluded from both multivariable models to avoid multicollinearity.

## Discussion

Notably, this study evaluated patients treated exclusively within the Brazilian public health system (*Sistema Único de Saúde*, SUS). Regarding the distribution of large B-cell lymphomas in the current cohort, PMBCL accounted for 7.3% (22/301) of the total cases, with DLBCL-NOS comprising the remaining 92.7% (279/301). This frequency is highly consistent with published epidemiological data, where PMBCL typically represents approximately 7% of diffuse large B-cell lymphoma presentations and 2%−4% of all non-Hodgkin lymphomas [7]. However, a high frequency of advanced-stage disease (Stage III–IV) was observed in 69.7% (210/301) of cases, whereas international studies report this proportion to be between 55% and 59%. Another noteworthy finding was the elevated rate of extranodal involvement, identified in 250 cases (83%), compared to international rates ranging from 25%−36% [7,35,37]. Additionally, a high proportion of patients presenting with high-risk features was observed; specifically, those classified into the R-IPI 'poor' risk category (≥3) and the NCCN-IPI high-intermediate/high-risk groups (≥4) accounted for 51.8% and 52.2% of the cohort, respectively. In comparison, international studies report these rates to be between 34% and 45% [7,35,37]. National data were consulted to better understand these discrepancies. The Brazilian study by Lage et al. [49], conducted in the same institution between 2009 and 2020, analyzed elderly patients (≥70 years) and reported similar findings: 72.4% of patients presented with advanced-stage disease and 76.2% had extranodal involvement. These findings support the hypothesis that patients treated within SUS may be more frequently diagnosed at advanced stages of disease. Although these findings suggest a higher disease burden in this Brazilian cohort, similar outcomes were observed on analyzing outcomes in DLBCL-NOS stratified by NCCN-IPI risk groups.

Higgins et al. [50] analyzed 51 cases of PMBCL and reported CD30 positivity in 69% of patients. In contrast, CD30 expression was identified in only 27.3% (6/22) of PMBCL cases in the present study. Five of these cases showed expression in >40% of tumor cells. One possible explanation for this discrepancy may lie in the type of sample used. Only three cases (13.6%) were assessed through excisional biopsies. Nevertheless, within the DLBCL-NOS subgroup, the frequency of CD30 expression was 19.0% using a positivity threshold of >0% and 11.0% when applying a cutoff of >20%. These values are highly consistent with the ranges reported in the literature. However, considerable variability has been observed across studies, which may be attributed to differences in sample sizes and the cutoff values used to define positivity. For example, Saputra et al. reported a CD30 positivity rate of 7% using a > 20% cutoff [51], whereas Wang et al., using the same criterion, observed a significantly higher rate of 22% [32]. Furthermore, studies such as those by Slack et al., Wang et al., and Saputra et al. demonstrated marked variations in CD30 expression rates depending on the cutoff applied (>0 vs. >20%): 25% vs. 12% [29], 41% vs. 22% [32], and 26% vs. 7% [51], respectively. It is noteworthy that, consistent with the present findings, the majority of studies have reported no significant association between CD30 expression and the cell-of-origin classification (GCB vs. non-GCB) [30,32,33,52]. Nevertheless, two studies identified a significant correlation between CD30 expression and the non-GCB subtype [29,34].

Because published studies evaluating the prognostic significance of CD30 expression have focused almost exclusively on DLBCL-NOS, this study restricted discussion to this specific subgroup. Using a CD30 positivity threshold of >0%, CD30 expression demonstrated no statistically significant association with either OS or PFS. These findings align with several published reports, yet contrast with others that attribute either a favorable or unfavorable prognostic significance to CD30 expression (Table 4). For example, Hu et al. analyzed a cohort of 903 patients with DLBCL and found that CD30 expression was associated with superior 5-year OS (79%vs. 59%; p = 0.001) and PFS (73%vs. 57%; p = 0.003) [27]. Conversely, Hao et al., in a study of 146 patients, identified CD30 expression as an unfavorable prognostic factor, with worse OS and PFS (5-year OS: 19.1% vs. 58%; p = 0.032; PFS: 12.9% vs. 58.4%; p = 0.041) [31]. Other studies, such as that of Wang et al., found no association between CD30 expression and prognosis. Similarly, Xu et al., evaluating patients treated with R-EPOCH [49] reported no significant impact of CD30 expression on OS [29]. Studies by Slack et al. [29] and Gong et al. [33] support the present findings, as they also did not identify a significant prognostic association with CD30 expression.

**Table 4:** Prognostic impact of CD30 expression in DLBCL across published studies and the present cohort.

Study reference (country)	OS		PFS		Notes
	(CD30⁺ vs. CD30⁻) HR (95% CI:)	p-value	(CD30⁺ vs. CD30⁻) HR (95% CI:)	p-value	
Hu et al., 2013 [27] (USA) n = 903	0.33 (0.15–0.75)	0.0082	0.35 (0.16–0.74)	0.0064	CD30-positive associated with better prognosis
Slack et al., 2014 [29] (Canada) n = 385		0.154		0.088	Non-significant difference
Hao et al., 2015 [31] n = 146	4.71 (1.96–11.30)	0.001	3.39 (1.56–7.38)	0.002	CD30-positive associated with worse prognosis
Gong et al., 2015 [30] (China) n = 232	—	0.049	—	0.029	Significant only in R-CHOP group
Wang et al., 2016 [32] (USA) n = 98	—	>0.05	—	>0.05	Non-significant difference
Xu et al., 2016 [49] (USA) n = 97		>0.05		>0.05	Non-significant difference
Gong et al., 2018 [33] (China) n = 241	0.45 (0.11–1.94)	0.284	0.44 (0.13–1.47)	0.182	Non-significant difference
Salas et al., 2020 [34] (Spain) n = 216	—	—	0.60 (0.30–1.00)	0.08	Non-significant difference
Present Study (Brazil) n = 279	0.76 (0.43–1.35)	0.35	0.91 (0.54–1.51)	0.703	Non-significant difference

DLBCL-NOS: Diffuse large B-cell lymphoma not otherwise specified; OS: Overall survival; PFS: Progression-free survival.

CD30 likely identifies a biologically distinct DLBCL subset, but its isolated prognostic value is limited by biological heterogeneity, technical variability, and confounding clinical factors. Future research should explore the circulating soluble form of CD30 as a potential biomarker of systemic activation of the TNF/CD30–CD30L pathway, correlating serum levels with treatment response, relapse, and tumor immune microenvironment features. Integrative studies combining serum biomarkers, tissue CD30 expression, and genomic or transcriptomic profiling (e.g., NF-κB and apoptotic pathway analyses) may help determine whether CD30 represents merely a phenotypic activation marker or plays a regulatory role in DLBCL pathobiology.

## Conclusion

In this study, CD30 expression was observed in 19.6% of 301 cases of DLBCL-NOS and PMBCL, with a median expression of 50% in neoplastic tissue (range: 5%–100%). The frequency of CD30 expression was 19% in DLBCL-NOS and 27.3% in PMBCL cases. No prognostic impact of CD30 expression was identified. Additionally, no significant associations were found between CD30 expression and NCCN-IPI and cell of origin classification. Multivariable analysis of the 301-patient cohort revealed that independent risk factors for reduced OS and PFS included age >60 years, the presence of B symptoms, and advanced Lugano stage (III-IV). These findings remained consistent when specifically analyzing the subgroup of 279 DLBCL-NOS cases.

## Data availability

The data that support the findings of this study are available from the corresponding author upon reasonable request.

## Conflicts of interest

None

## Appendix Supplementary materials

Image, application 1
