# Supplementary material for: CD30 expression in diffuse large B-cell lymphoma and primary mediastinal lymphoma: frequency, association with prognostic factors, and impact on survival
Source: Hematol Transfus Cell Ther. 2026 Aug 5;48(4):106493. doi: 10.1016/j.htct.2026.106493 (PMC13474331; doi:10.1016/j.htct.2026.106493)
Supplement: Supplementary file 1 [file mmc1.docx]

**Supplementary Material**

The supplement presents the data, univariate and multivariate Cox analyses of Progression-free Survival (PFS) and Overall Survival (OS) for all patient cohort and for the Diffuse Large B-Cell Lymphoma, Not Otherwise Specified (DLBCL, NOS) subgroup.

Table 1: Distribution of factors associated with Progression-Free Survival for all patients: Univariate Cox Analysis

| **Covariate** | **Level** | **N** | **HR(95% CI)** | **P-value** |
| --- | --- | --- | --- | --- |
| Disease | PML^1^ | 22 | 0.57 (0.23-1.41) | 0.224 |
|  | DLBCL,NOS^2^ | 278 | - | **-** |
|  | | | | |
| Cell-of-origin classification | Germinal center | 140 | 0.79 (0.54-1.17) | 0.239 |
|  | Non-germinal center | 160 | - | **-** |
|  | | | | |
| CD30 expression | >0 (positive) | 59 | 0.81 (0.49-1.35) | 0.416 |
|  | 0 (negative) | 241 | - | **-** |
|  | | | | |
| Gender | Male | 147 | 1.10 (0.75-1.61) | 0.617 |
|  | Female | 153 | - | **-** |
|  | | | | |
| Age (in Years) | >60 | 133 | 1.76 (1.20-2.59) | **0.004** |
|  | ≤ 60 | 167 | - | **-** |
|  | | | | |
| B symptoms | Present | 207 | 2.08 (1.29-3.37) | **0.003** |
|  | Absent | 90 | - | **-** |
|  | | | | |
| Lugano Staging | III-IV | 210 | 2.26 (1.37-3.71) | **0.001** |
|  | I-II | 88 | - | **-** |
|  | | | | |
| ECOG | ≥2 | 76 | 1.89 (1.27-2.83) | **0.002** |
|  | 0-1 | 222 | - | **-** |
|  | | | | |
| Extranodal involvement | 1:Yes | 249 | 0.97 (0.60-1.59) | 0.919 |
|  | 0:No | 51 | - | **-** |
|  | | | | |
| LDH^3^ | Elevated | 182 | 1.90 (1.26-2.89) | **0.002** |
|  | Normal | 118 | - | **-** |
|  | | | | |
| NCCN-IPI^4^ | ≥4 | 157 | 2.91 (1.91-4.44) | **<.001** |
|  | <4 | 143 | - | **-** |
|  | | | | |
| IPI^5^ | ≥ 3 | 156 | 2.47 (1.64-3.71) | **<.001** |
|  | <3 | 144 | - | **-** |
|  | | | | |
| Regimen treatment | R-CHOP^6^ | 271 | 2.33 (0.95-5.72) | 0.064 |
|  | others | 29 | - | **-** |
|  | | | | |

1: PML: Primary Mediastinal Lymphoma; 2: Diffuse Large B Cell Lymhpoma Not Otherwise Specified; 3: LDH: Lactate dehydrogenase; 4: NCCN-IPI: National Comprehensive Cancer Network International Prognostic Index; 5: IPI: International Prognostic Index 6: RCHOP: Rituximab cyclophosphamide, doxorubicin, vincristine, and prednisone HR (95% CI): Crude Hazard Ratio with 95% Confidence Interval

Table 2: Multivariate Cox Regression Analysis with Backward Selection for Progression-Free Survival in the total cohort

| **Covariate** | **Level** | **aHR(95% CI)** | **P-value** |
| --- | --- | --- | --- |
| Age (in years) | >60 | 1.70 (1.15-2.50) | **0.007** |
|  | ≤60 | - | - |
|  | | | |
| B symptoms | Present | 1.82 (1.12-2.97) | **0.016** |
|  | Absent | - | - |
|  | | | |
| Lugano Staging | III-IV | 2.04 (1.24-3.38) | **0.005** |
|  | I-II | - | - |
|  | | | |
| *  Number of observations used = 295. ** Backward selection with an alpha level of removal of 0.05 was used. The following variables were removed from the model: LDH, ECOG, IPI, Regimen Treatment. NCCN-IPI was not considered due to multicollinearity | | | |

aHR (95% CI): Adjusted Hazard Ratio with 95% Confidence Interval

| **Covariate** | **Level** | **N** | **HR(95% CI)** | **P-value** |  |  |
| --- | --- | --- | --- | --- | --- | --- |
| Disease | PML^1^ | 22 | 0.39 (0.12-1.22) | 0.105 |  |  |
|  | DLBCL, NOS^2^ | 278 | - | **-** |  |  |
|  | | | | |  |  |
| Cell-of-origin classification | Germinal center | 140 | 0.78 (0.52-1.18) | 0.245 |  |  |
|  | Non-germinal center | 160 | - | **-** |  |  |
|  | | | | |  |  |
| CD30 expression | >0 (positive) | 59 | 0.70 (0.40-1.24) | 0.219 |  |  |
|  | 0 (negative) | 241 | - | **-** |  |  |
|  | | | | |  |  |
| Gender | Male | 147 | 1.10 (0.73-1.65) | 0.662 |  |  |
|  | Female | 153 | - | **-** |  |  |
|  | | | | |  |  |
| Age (in years) | >60 | 133 | 1.96 (1.29-2.96) | **0.001** |  |  |
|  | ≤ 60 | 167 | - | **-** |  |  |
|  | | | | |  |  |
| B symptoms | Yes | 207 | 2.08 (1.24-3.49) | **0.006** |  |  |
|  | No | 90 | - | **-** |  |  |
|  | | | | |  |  |
| Lugano Staging | III-IV | 210 | 2.70 (1.53-4.78) | **<.001** |  |  |
|  | I-II | 88 | - | **-** |  |  |
|  | | | | |  |  |
| ECOG | ≥2 | 76 | 2.00 (1.31-3.07) | **0.001** |  |  |
|  | 0-1 | 222 | - | **-** |  |  |
|  | | | | |  |  |
| Extranodal involvement | Yes | 249 | 1.02 (0.60-1.72) | 0.955 |  |  |
|  | No | 51 | - | **-** |  |  |
|  | | | | |  |  |
| LDH^3^ | Elevated | 182 | 1.96 (1.25-3.07) | **0.003** |  |  |
|  | Normal | 118 | - | **-** |  |  |
|  | | | | |  |  |
| NCCN-IPI^4^ | ≥4 | 157 | 3.20 (2.01-5.07) | **<.001** |  |  |
|  | <4 | 143 | - | **-** |  |  |
|  | | | | |  |  |
| IPI^5^ | ≥ 3 | 156 | 2.82 (1.80-4.42) | **<.001** |  |  |
|  | <3 | 144 | - | **-** |  |  |
|  | | | | |  | |
| Regimen treatment | R-CHOP^6^ | 271 | 1.93 (0.78-4.75) | 0.153 |  |  |
|  | others | 29 | - | **-** |  |  |
|  | | | | |  |  |

Table 3: Distribution of factors associated with Overall Survival for all patients: Univariate Cox Analysis

1:PML Primary Mediastinal Lymphoma; 2: Diffuse Large B Cell Lymhpoma ; 3: LDH: Lactate dehydrogenase; 4: NCCN-IPI: National Comprehensive Cancer Network International Prognostic Index; 5: IPI: International Prognostic Index 6: RCHOP: Rituximab cyclophosphamide, doxorubicin, vincristine, and prednisone

Table 4: Multivariate Cox Regression Analysis with Backward Selection for Overall Survival in the total cohort

| **Covariate** | **Level** | **HR(95% CI)** | **P-value** |
| --- | --- | --- | --- |
| Age (in years) | >60 | 1.89 (1.24-2.87) | **0.003** |
|  | <=60 | - | - |
|  | | | |
| B symptoms | Present | 1.75 (1.03-2.95) | **0.037** |
|  | Absent | - | - |
|  | | | |
| Lugano Staging | III-IV | 2.45 (1.38-4.36) | **0.002** |
|  | I-II | - | - |
|  | | | |
| *  Number of observations used = 295. ** Backward selection with an alpha level of removal of 0.05 was used. The following variables were removed from the model: LDH, ECOG, IPI, Disease. NCCN-IPI were not considered due to multicollinearity | | | |

| **Covariate** | **Level** | **N** | **HR(95% CI)** | **P-value** |
| --- | --- | --- | --- | --- |
| Cell-of-origin classification | Germinal center | 132 | 0.77 (0.52-1.14) | 0.189 |
|  | Non-germinal center | 146 | - | **-** |
|  | | | | |
| CD30 expression | >0 (positive) | 53 | 0.91 (0.54-1.51) | 0.703 |
|  | 0 (negative) | 225 | - | **-** |
|  | | | | |
| Gender | Male | 135 | 1.10 (0.74-1.62) | 0.636 |
|  | Female | 143 | - | **-** |
|  | | | | |
| Age (in years) | >60 | 130 | 1.61 (1.09-2.39) | **0.017** |
|  | ≤ 60 | 148 | - | **-** |
|  | | | | |
| B symptoms | Yes | 188 | 2.21 (1.35-3.62) | **0.002** |
|  | No | 87 | - | **-** |
|  | | | | |
| Lugano Staging | III-IV | 202 | 2.80 (1.59-4.92) | **<.001** |
|  | I-II | 75 | - | **-** |
|  | | | | |
| ECOG | ≥2 | 73 | 1.99 (1.32-2.98) | **<.001** |
|  | 0-1 | 203 | - | **-** |
|  | | | | |
| Extranodal involvement | Yes | 237 | 1.04 (0.61-1.77) | 0.893 |
|  | No | 41 | - | **-** |
|  | | | | |
| LDH^1^ | Elevated | 165 | 1.87 (1.23-2.85) | **0.004** |
|  | Normal | 113 | - | **-** |
|  | | | | |
| NCCN-IPI^2^ | ≥4 | 150 | 3.12 (2.01-4.86) | **<.001** |
|  | <4 | 128 | - | **-** |
|  | | | | |
| IPI^3^ | ≥ 3 | 150 | 2.72 (1.77-4.19) | **<.001** |
|  | <3 | 128 | - | **-** |
|  | | | | |
| Regimen treatment | R-CHOP^5^ | 258 | 1.62 (0.66-3.97) | 0.296 |
|  | others | 20 | - | **-** |

Table 5: Distribution of factors associated with Progression-Free Survival for Diffuse Large B cell Lymphoma, Not Otherwise Specified cohort: Univariate Cox Analysis

1: LDH: Lactate dehydrogenase; 2: NCCN-IPI: National Comprehensive Cancer Network International Prognostic Index; 3: IPI: International Prognostic Index; 4: CHOP: Rituximab cyclophosphamide, doxorubicin, vincristine, and prednisone

Table 6: Multivariate Cox Regression Analysis with Backward Selection for Progression-Free Survival in for Diffuse Large B cell Lymphoma, Not Otherwise Specified cohort

| **Covariate** | **Level** | **HR(95% CI)** | **P-value** |
| --- | --- | --- | --- |
| Age (in years) | >60 | 1.57 (1.05-2.34) | **0.026** |
|  | ≤60 | - | - |
|  | | | |
| B symptoms | Present | 1.87 (1.14-3.08) | **0.014** |
|  | Absent | - | - |
|  | | | |
| Lugano staging | III-IV | 2.53 (1.43-4.47) | **0.001** |
|  | I-II | - | - |
| * Number of observations used = 274. ** Backward selection with an alpha level of removal of 0.05 was used. The following variables were removed from the model: LDH, ECOG, IPI. NCCN-IPI was not considered due to multicollinearity | | | |

Table 7: Distribution of factors associated with Overall Survival for Diffuse Large B cell Lymphoma, Not Otherwise Specified cohort: Univariate Cox Analysis

| **Covariate** | **Level** | **N** | **HR(95% CI)** | **P-value** |
| --- | --- | --- | --- | --- |
| Cell-of-origin classification | germinal center | 132 | 0.72 (0.48-1.10) | 0.131 |
|  | non-germinal center | 146 | - | **-** |
|  | | | | |
| CD30 expression | >0 | 53 | 0.76 (0.43-1.35) | 0.350 |
|  | 0 | 225 | - | **-** |
|  | | | | |
| Gender | 1:masculino | 135 | 1.03 (0.68-1.56) | 0.876 |
|  | 0:feminino | 143 | - | **-** |
|  | | | | |
| Age (in years) | >60 | 130 | 1.79 (1.17-2.72) | **0.007** |
|  | ≤60 | 148 | - | **-** |
|  | | | | |
| B symptoms | Present | 188 | 2.27 (1.34-3.87) | **0.002** |
|  | Absent | 87 | - | **-** |
|  | | | | |
| Lugano Staging | III-IV | 202 | 3.11 (1.65-5.85) | **<.001** |
|  | I-II | 75 | - | **-** |
|  | | | | |
| ECOG | ≥2 | 73 | 2.05 (1.33-3.15) | **0.001** |
|  | 0-1 | 203 | - | **-** |
|  | | | | |
| Extranodal involvement | Yes | 237 | 1.04 (0.59-1.85) | 0.884 |
|  | No | 41 | - | **-** |
|  | | | | |
| LDH^1^ | Elevated | 165 | 1.97 (1.25-3.10) | **0.003** |
|  | Normal | 113 | - | **-** |
|  | | | | |
| NCCN-IPI^2^ | ≥4 | 150 | 3.40 (2.10-5.52) | **<.001** |
|  | <4 | 128 | - | **-** |
|  | | | | |
| IPI^3^ | ≥3 | 150 | 2.97 (1.86-4.74) | **<.001** |
|  | <3 | 128 | - | **-** |
|  | | | | |
| Regimen treatment | R-CHOP^4^ | 258 | 1.35 (0.55-3.34) | 0.511 |
|  | Others | 20 | - | **-** |
|  | | | | |

1: LDH: Lactate dehydrogenase; 2: NCCN-IPI: National Comprehensive Cancer Network International Prognostic Index; 3: IPI: International Prognostic Index; 4: CHOP: Rituximab cyclophosphamide, doxorubicin, vincristine, and prednisone

Table 8: Multivariate Cox Regression Analysis with Backward Selection for Overall Survival in for Diffuse Large B cell Lymphoma, Not Otherwise Specified cohort

| **Covariate** | **Level** | **HR(95% CI)** | **P-value** |
| --- | --- | --- | --- |
| Age (in years) | >60 | 1.74 (1.14-2.68) | **0.011** |
|  | ≤60 | - | - |
|  | | | |
| B symptoms | Present | 1.86 (1.08-3.18) | **0.025** |
|  | Absent | - | - |
|  | | | |
| Lugano staging | III-IV | 2.81 (1.48-5.33) | **0.002** |
|  | I-II | - | - |
|  | | | |
| *   Number of observations used = 274. ** Backward selection with an alpha level of removal of 0.05 was used. The following variables were removed from the model: Cell-of-origin classification, LDH, ECOG and IPI. NCCN-IPI was not considered due to multicollinearity | | | |
